# Asymptomatic Malaria and its Challenges in the Malaria Elimination Program in Iran: a Systematic Review

**Published:** 2017-05-27

**Authors:** Gholmreza Hassanpour, Mehdi Mohebali, Hojjat Zeraati, Ahmad Raeisi, Hossein Keshavarz

**Affiliations:** 1 Center for Research of Endemic Parasites of Iran, Tehran University of Medical Sciences, Tehran, Iran; 2 Department of Medical Parasitology and Mycology, School of Public Health, Tehran University of Medical Sciences, Tehran, Iran; 3 Department of Epidemiology and Biostatistics, School of Public Health, Tehran University of Medical Sciences, Tehran, Iran

**Keywords:** Malaria, Asymptomatic infection, Elimination

## Abstract

**Background:**

The objective of this study was to find an appropriate approach to asymptomatic malaria in elimination setting through a systematic review.

**Methods:**

A broad search was conducted to find articles with the words ‘malaria’ in their titles and ‘asymptomatic’ or ‘submicroscopic’ in their texts, irrespective of the type of study conducted. The Cochrane, Medline/Pub Med, and Scopus databases, as well as Google Scholar were systematically searched for English articles and reports and Iran’s databases-Iran Medex, SID and Magiran were searched for Persian reports and articles, with no time limitation. The study was qualitatively summarized if it contained precise information on the role of asymptomatic malaria in the elimination phase.

**Results:**

Six articles were selected from the initial 2645 articles. The results all re-emphasize the significance of asymptomatic malaria in the elimination phase, and emphasize the significance of diagnostic tests of higher sensitivity to locate these patients and perform interventions to reduce the asymptomatic parasitic reservoirs particularly in regions of low transmission. However, we may infer from the results that the current evidence cannot yet specify an accurate strategy on the role of asymptomatic malaria in the elimination phase.

**Conclusion:**

To eliminate malaria, alongside vector control, and treatment of symptomatic and asymptomatic patients, active and inactive methods of case detection need to be employed. The precise monitoring of asymptomatic individuals and submicroscopic cases of malaria through molecular assays and valid serological methods, especially in regions where seasonal and low transmission exists can be very helpful at this phase.

## Introduction

Across the globe, 3.3 billion people are at risk of malaria. In 2013, 198 million people were affected with the disease, of which 584000 people died (World Health Organization 2014). A 50% reduction in the number of malaria cases by 2015 was announced as a Millennium Developmental Goal. Currently, 99 countries are malaria-free and 34 countries are in the phase of elimination (Gueye et al. 2013). The countries that are in the elimination phase have put different strategies on their agendas, such as general screening and focusing on specific foci (World Health Organization 2013). In such countries, at the same time that transmission reduces, the major share of transmission will take place through collective and focal transmission (Ministry of Health and Medical Education 2012).

Probably, the infected asymptomatic individual can become a source of parasitic transmission to healthy individuals under favorable setting, considered a serious challenge to malaria control and elimination worldwide (Lin et al. 2014). The popular belief is that in low transmission settings the proportion of asymptomatic individuals is less than that in areas of greater transmission severity. However, community-based studies have shown that, although an increased transmission state is associated with an increase in the reservoir share, but even in low transmission areas the asymptomatic cases make up for 60% of the infected population (Sturrock et al. 2013). Therefore, it seems that in low transmission settings the malaria infection is very likely to be asymptomatic (WHO 2013). While symptomatic cases are removed from the disease reservoir faster because of treatment. Under such circumstances, communities appear to need interventions that address beyond symptoms (Sturrock et al. 2013).

Under circumstances in which many successful and unsuccessful attempts at malaria elimination have been made worldwide, this review was designed to find an appropriate strategy for the detection and elimination of asymptomatic malaria. The summarization of these experiences in the form of a systematic review can effectively help clarify these experiences and in turn help national policymakers who are trying to eliminate malaria in Iran

## Materials and Methods

### Study design

This study has systematically searched all the studies, reports and documentations related to malaria and the role of asymptomatic malaria in the elimination phase-with no time limitation-up to July 2015.

### Inclusion criteria

In this systematic review, all the articles that had questioned or responded to the main question of the study, i.e. ‘the role and significance of asymptomatic malaria cases in the pre-elimination and elimination phases of malaria’ irrespective of the study design-were selected.

### Search strategy

The Cochrane, Medline/PubMed, and Scopus databases, as well as Google Scholar were systematically searched for English articles and reports and Iran’s databases-IranMedex, SID and Magiran were searched for Persian reports and articles, with no time limitation. We performed a search of high sensitivity to find the relevant articles. In short, “(Malaria in Title) AND (asymptomatic OR sub microscopic OR sub-microscopic OR low parasite) were searched in various databases.

Selecting studies, quality assessment and data extraction all the article titles were first entered into reference manager software (End-Note). After omitting the duplicate cases, they were prepared for the initial screening. In the first stage, the article titles were reviewed and those articles that were obviously irrelevant to the study objective were removed from the primary databank. In the next stage, two persons studied the titles and abstracts and the articles that were selected by both persons were finalized. If only one person chose an article, then that article was discussed in a meeting and the decision to include or exclude it in the study was made thereafter. At this stage, the selected articles’ full texts were obtained and those articles that had specifically answered the following two questions were selected as candidates for inclusion. The two questions were, “Has the article discussed the topic of Asymptomatic and/or Submicroscopic malaria? Have these topics been applicable in the elimination phase of malaria?” If the answers to these questions were ‘yes’ then the article would be included in the study. The selected studies were thoroughly reviewed by two persons and their opinions were shared with each other in a joint meeting. If the overall impression of the applicability of the study results in malaria elimination was the same then that article has been selected.

## Results

**Fig. 1.**
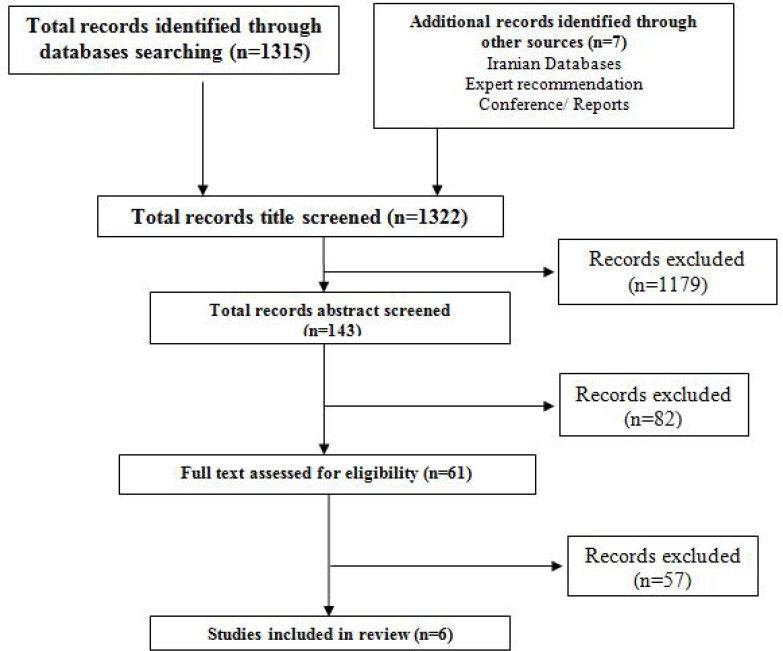
Flow chart demonstrating database searches, identification, screening and selection of included studies

The systematic review yielded 2645 articles in all (Medline/PubMed: 718, Magiran: 210, IranMedex: 226, SID: 74, Web of Science: 668, Scopus: 748, other databases such as EMB Reviews and Cochrane: 1). After omitting the duplicate cases 1322 articles were sorted for title review. Upon reviewing the article titles, 143 articles, and upon reviewing the abstracts 61 articles remained in the databank, respectively. The full texts of these 61 articles were obtained and eventually, upon studying those, only six articles were finalized (Fig. 1, Table 1). The most important reason for exclusion in the final stage was lack of a direct relation and an applicable result in malaria elimination settings.

**Table 1.** Characteristics of included studies

**Author**	**Year**	**Type of study**	**Aim**	**Recommended policy**	**Challenges/ acceptability**
**Bousema et al.**	2014	Opinion	Asymptomatic malaria and public health relevance	Interventions should target both symptomatic and asymptomatic individuals, depending on the circumstances	Can molecular methods employed to detect asymptomatic individuals help reduce transmission?
**Lindblade et al.**	2013	Review	The role of asymptomatic malaria in malaria transmission and interventions to achieve malaria elimination	By choosing efficient interventions for asymptomatic patients, parasitic reservoirs should be destroyed.	Lack of a unified definition of asymptomatic malaria
**Lin et al.**	2014	Review	The role of submicroscopic parasitemia in malaria transmission	Advising molecular diagnostic tests in longitudinal studies and exploring the role of reservoirs in malaria transmission	Absence of scientific evidence supporting or opposing the role of submicroscopic malaria in transmission
**Sturrock et al.**	2013	Policy forums	Potential role of ACD in malaria control and elimination	RACD appropriate to low-transmission settings and PACD appropriate to low/medium transmission	There is no evidence of the cost-effectiveness of these interventions in the long run
**Malera consultive group**	2011	Review	To propose a research agenda for the tools required for malaria eradication	The need for diagnostic tests with little parasitic density in asymptomatic persons	Difficulties are observed in countries with little resources; also the companies that can tend to these priorities are small companies that need support
**Moonen et al.**	2010	Opinion	To review the activities needed to achieve and maintain malaria elimination	Establishment of a surveillance system connected to rapid response surveillance over time	There is no the global campaign, national elimination cannot succeed with continued importation.

In summary, the selected articles emphasized the significance of the role of asymptomatic malaria in the elimination phase. They also emphasized the need for diagnostic tests of higher sensitivity to detect these cases and the performance of interventions aimed at reducing parasite reservoirs-with an emphasis on asymptomatic cases.

Lind blade et al. published an article on the significance of asymptomatic malaria in 2013. They stated that although malaria control has had many positive effects in the world, there is not much certainty regarding the current interventions aimed at eliminating malaria in many endemic countries. In this review, a great share of the infected cases of malaria has been attributed to asymptomatic individuals who were not seeking treatment. These circumstances become even more challenging in regions where *Plasmodiumvivax* contributes more to the burden of disease because of its dormant liver stage. They conclude by stating the important role asymptomatic malaria has to play in its transmission and that in order to eliminate the disease, interventions must target the reduction of parasite reservoirs in both low and high transmission setting (Sturrock et al. 2013).

“Targeting asymptomatic malaria infections: active surveillance in control and elimination”-another study published in 2013 by Sturrock et al. underscores that scale up malaria control programs in various countries have reduced the focal transmission of disease. In a smaller scale, each focus consists of hotspots such as households and groups of households with higher malaria transmission and consistent parasite environments throughout the year. This infection is clustered in specific populations (hotspots) that have high-risk demographic characteristics. The authors believe that the identification of these groups-both geographically and demographically-is an important strategy toward reducing local parasite reservoirs and disrupting the transmission chain in low transmission or elimination setting (Ministry of Health and Medical Education 2012)

The current approaches toward malaria elimination are based on the recommendations of the Global Malaria Eradication Program of 1960. However, many countries face multiple challenges in the fourth phase of elimination, such as, the imported cases of infection, which apparently requires a regional approach. To eliminate malaria, focus needs to be laid on the detection and elimination of infectious foci through active and inactive methods of patient detection. In this approach, alongside vector control, both symptomatic and asymptomatic patients should be treated correctly. Since imported cases can be a source of disease transmission, malaria elimination programs should have precise plans for these cases as well (Moonen et al. 2010).

In countries that have gone through the control phase, to ensure malaria elimination and monitor transmission reduction, diagnostic tools applicable in the field are necessary to detect asymptomatic and low-density parasitic cases for extensive screening and treatment (Lo et al. 2015).

In an article Lin et al. (2014) emphasized that malaria elimination can be achieved by eliminating the infected human reservoirs (including asymptomatic cases). A better understanding of the role of submicroscopic cases as a source of transmission can greatly help to adopt an active surveillance approach. According to this article, the results of screening have shown the capability of RDTs in detecting asymptomatic cases at a threshold of 200 parasites per microliter, which is a submicroscopic threshold. Therefore, it seems that in order to eliminate, we need newer methods of higher sensitivity to be able to detect potential transmission agents at any scale (World Health Organization 2013).

Molecular diagnostic tests have shown that even in low endemic regions, asymptomatic malaria reservoirs are more than they were believed to be. Individuals that can be diagnosed with microscopic or submicroscopic methods have submicroscopic gametocyte density. Bousema et al. (2014) believed that, in order to eliminate malaria, interventions should target both symptomatic and asymptomatic infections. In regions where there is seasonal and low transmission of malaria, even a small percentage of infected persons are sufficient to re-start malaria transmission. Depending on various conditions, the transmission of infection from mosquito to human is different, however, as a barrier to malaria elimination, asymptomatic and submicroscopic cases should be monitored through appropriate molecular methods (Lindblade et al. 2013).

## Discussion

We found only a few studies with goals completely relevant to our research question. None of the studies had discussed with certainty the role of asymptomatic malaria and the correct approach toward these cases, particularly in malaria elimination programs. The evidence suggested that asymptomatic malaria probably has a major role to play in malaria transmission. Communities need tools of higher sensitivity to detect asymptomatic and low-parasitic density cases. Malaria elimination requires interventions aimed at reducing parasite reservoirs, both in high- and low-transmission setting.

In this systematic review, we tried to retrieve all the relevant data. However, most of the selected articles including review articles were expert commentaries and recommendations. However, the scientific community cannot cite such evidence with great certainty when it comes to the evidence pyramid. The lack of interventions and prospective cohort studies relevant to the research question on one hand, and the high significance of climate, parasitic species and vector differences on the other, does not allow a conclusion to be reached with certainty on the strategy of dealing with asymptomatic patients-particularly at the elimination phase of the disease. Although experts have emphasized the role of asymptomatic carrier in various studies, but the interesting point is the absence of a unified definition of asymptomatic malaria (Moonen et al. 2010, Hassanpour et al. 2011).

Most studies have described asymptomatic malaria by the presence of sexual or asexual multiplication parasites and/or absence of acute clinical symptoms (usually fever) during a specific period. Some studies have focused on the parasite density threshold, and have considered febrile patients with higher than pre-determined threshold parasite densities symptomatic. Various studies have set different criteria and given different definitions for being symptomatic, such as, different follow-up periods for becoming symptomatic, taking into account one symptom (mostly fever) and/or all clinical symptoms (Sturrock et al. 2013). Perhaps the first shortcoming in adopting an operational approach toward asymptomatic malaria is the lack of a unified definition of asymptomatic malaria in different references.

Asymptomatic malaria can become symptomatic due to relative immunity, frequency of exposure to malaria infection, increased age (independent of repeated exposure), and cross-protection resulting from relative immunity from exposure to multi clonal infections. Among various factors, age and previous exposure are the most important determinant factors of immunity in individuals (Sturrock et al. 2013).

Moreover, sometimes, symptomatic persons who have not been treated enter the chronic phase of the disease and reach a parasite level lower than threshold-which is not observable under the microscope. Another important group are those persons who have recently received treatment or have been diagnosed early (before becoming symptomatic) following a diagnostic test such as PCR, and/or have a parasite density level that has never become observable under the microscope (Lindblade et al. 2013). Hence, we may conclude that asymptomatic malaria includes many different types of patients.

In addition to these groups, we must keep in mind that some asymptomatic individuals are patients who have received treatment in the past, those who have probably appeared asymptomatic because of incomplete treatment or relative drug resistance. This matter requires meticulous studies to identify the genotype of the parasite, in terms of whether it is a residual infection or a new one. Nevertheless, in addition to the aforementioned complexities, associated infections with parasites such as *Ascaris lumbricoides* and *Schistosomia hematobium* too can complicate the detection of these individuals because of cross reactivity (Sturrock et al. 2013).

Evidence suggests that all species of malaria can result in asymptomatic disease. However, since *P. falciparum* and *P. vivax* are more prevalent, most asymptomatic patients are infected with these two *Plasmodium* species. The *P. vivax* species create immunity more rapidly as compared to falciparum species, hence raising the possibility of controlling the parasite density as well. Although there are fewer *P. vivax* studies than there are *P. falciparum* ones, but it seems that in this species too, an increase in the number of gametes raises the possibility of the insect’s infectivity. Various studies in different settings and with different diagnostic tools have shown entirely variable results, such that, we cannot draw an association between the asymptomatic cases of malaria and its different species (Sturrock et al. 2013).

Another challenge in approaching asymptomatic malaria is the existent knowledge of the true role of the affected individuals in disease transmission. The uncertainty surrounding the role of submicroscopic malaria in disease transmission can be dangerous from two aspects. On one hand, asymptomatic malaria is considered a serious challenge in malaria control and elimination in many parts of the world, as it can under favorable conditions act as a reservoir and cause parasite transmission. On the other hand, if these cases are not treated effectively then they can provoke the global drug resistance problem (World Health Organzation 2013, Lin et al. 2014). Many believe that some of these individuals eventually become symptomatic. However, there is no certainty as to whether these individuals have been affected with a new febrile illness or that re-infection has taken place (Sturrock et al. 2013).

In addition to all the aforementioned diagnostic issues and reasons of presentation of these cases, the important question is the Infectivity of asymptomatic individuals. The general belief is that gametocyte density is the most important determinant factor of infection transmission from an infected individual to an anopheles vector (Diagnoses. 2011).

So, the lower the gametocyte carriage density, the less significant the issue of infectivity. Theoretically speaking, the possibility of transmission exists with even a single gametocyte per microliter. However, the current evidence does not point to a robust relationship between gametocyte density and asymptomatic individuals’ infectivity. Presently, it may be said that the gametocyte is an incomplete surrogate for transmission. The evidence on gametocyte carriage among asymptomatic cases is also insufficient. Direct skin test feeding, observance of oocysts in the *Anopheles* midgut, and membrane feeding apparatus studies may to some extent answer these questions. In addition to gametocyte density, other factors such as the vector and host also affect this transmission (Sturrock et al. 2013, World Health Organzation 2013).

Under the most favorable conditions and in standard laboratories, Giemsa-stained blood film optical microscopy can detect more than 10 parasites per microliter, while, ordinarily, approximately 100 parasites per microliter should be present in 5 microliters of blood. Methods such as qPCR have the ability to detect almost double the microscopic method, considering the groups at risk and the community in which the examination is being done (Lindblade et al. 2013). Depending on the diagnostic method, be it microscopy, RDT or PCR, the detection of asymptomatic cases has yielded completely different results in different communities (Haghi et al. 2012, Sturrock et al. 2013, Bousema et al. 2014).

The most important applied difference between control and elimination programs is the focus on detection of and intervention on symptomatic and asymptomatic infections (Moonen et al. 2010). As the prevalence of the disease falls, activities should focus on subclinical and low-density infections and reservoirs present in specific geographic and demographic areas. No doubt, to achieve the goals of disease elimination and eradication, we must be able to detect malaria infection with applicable diagnostic tests, something that is not possible with the current microscopic methods, where PCR is an expensive technique and not practical for the field (Laishram et al. 2012).

In low transmission or malaria elimination conditions, transmission takes place focally. Therefore, the approach of targeted detection of these infection clusters becomes extremely important for reducing local reservoirs and disrupting the transmission chain (Ministry of Health and Education 2012). Submicroscopic infections in high transmission regions (where parasite prevalence in the community exceeds 75% microscopically) are estimated at 20%, and at 70–80% in low transmission setting (where prevalence is less than 10%). Evidently, submicroscopic prevalence has increased in areas where control has been successful in reducing transmission (Lindblade et al. 2013). In low transmission setting, the share of asymptomatic cases in transmitting malaria is significant. In regions of seasonal transmission, they can be a source of infection for the new generations of mosquitoes following rain (Sturrock et al. 2013).

Active case detection is the intervention recommended for low malaria transmission settings, although there is very little evidence on its efficiency. The most important factors affecting the efficiency of this method are its low sensitivity, inability to detect very low-density infection, continuous importation of parasites, as well as insufficient coverage of the population (Ministry of Health and Medical Education 2012) .

The most important public health interventions aimed at asymptomatic parasitemia are ‘drug mediated strategies’ aimed at asymptomatic parasite reservoirs: 1-mass drug administration (MDA) in a geographic region-irrespective of infection and symptoms, 2-mass screening and treatment (MSAT) or aggressive active case detection, and 3-intermittent preventive therapy-repeated treatment of high-risk groups. These approaches can be adopted at smaller (modified) scales such as hotspots too. Mathematical models have shown a 15–20% reduction in transmission through the achievement of these goals (Sturrock et al. 2013).

To eliminate malaria, in addition to underscoring the significance of the highest public authorities’ commitment to the program, the World Health Organization has made the following recommendations:

Programs for detecting and treating patients, disrupting malaria transmission, identification of local foci, establishment of an active surveillance system, preventing re-establishment of transmission, and regional cooperation - particularly with neighboring countries to prevent the entry of infection into the country.

No doubt, the chances of eliminating malaria reduce if there are efficient mosquito vectors, special demographic conditions such as suburbanization, low hygiene levels, unsustainable borders and illegal migrations (Bousema et al. 2012).

As a country that has reduced approximately 90% of its cases, Iran is faced with the challenge of disease elimination due to multiple reasons. An important challenge is the issue of cross border importation from Afghanistan and Pakistan. 1.6% (7/446) asymptomatic Afghani immigrants are asymptomatic-infected who can be the potential source of parasites transmission (Nateghipour et al. 2011). Based on statistics, over 20% of the treated cases in Iran are patients from Pakistan, a country where there are 4.5 million reported cases of malaria, and from which the entries and exits into and from the Eastern borders are not meticulously controlled.

Moreover, in Iran, different vectors behave differently, which is another challenge of the elimination phase (Hanafi-Bojd et al. 2011). The resistance of the most important vector ie *Anopheles stephensi* to some insecticides, especially pyrethroids, (Vatandoost and Hanafi-Bojd 2012), as well as the drug resistance of *P. falciparum* to Chloroquine and some other drugs strongly necessitates an accurate monitoring to enable the achievement of the elimination program’s goals (Tietje et al. 2014).

## Conclusions

Detection of high-risk foci either it geographically or demographically, is an important strategy for reducing local parasite reservoirs and disrupting the transmission chain. Active and passive methods of case detection must be used to achieve the goal of malaria elimination. In this approach, both groups of clinically symptomatic and asymptomatic individuals need to be correctly diagnosed and treated, at the same time that vector control is under way. Imported malaria cases are a potential source of transmission. Thus, targeting symptomatic and asymptomatic infections particularly in regions of seasonal and low transmission, keeping in mind that even a small percentage of infected persons are sufficient to re-start the malaria transmission, demands the accurate monitoring of asymptomatic and submicroscopic cases through highly valid molecular and/or serological methods.

Iran is a country with a low burden and limited transmission of malaria, wherein the elimination of local malaria transmission from *P. falciparum* and the complete elimination of malaria have been targeted for 2015 and 2025, respectively. Most cases of disease however, result from *P. vivax*, which has special biological characteristics such as, the latent liver stage hypnozoites and the faster immunity created against it -compared to *P. falciparum*, thus necessitating the need to take into account other considerations as well (Lindblade et al. 2013). Conducting precise longitudinal follow-up studies on asymptomatic individuals, to examine the possibility of their being gametocytemic, and, to study the period of infection in these individuals in low transmission settings can yield more accurate data for correct planning by health policymakers.
